# Supplementary material for: The regulon of Brucella abortus two-component system BvrR/BvrS reveals the coordination of metabolic pathways required for intracellular life
Source: PLoS One. 2022 Sep 21;17(9):e0274397. doi: 10.1371/journal.pone.0274397 (PMC9491525; doi:10.1371/journal.pone.0274397)

S1_raw_images

Fig 1B, samples 1,2,11,12,15&16, photographed gel:

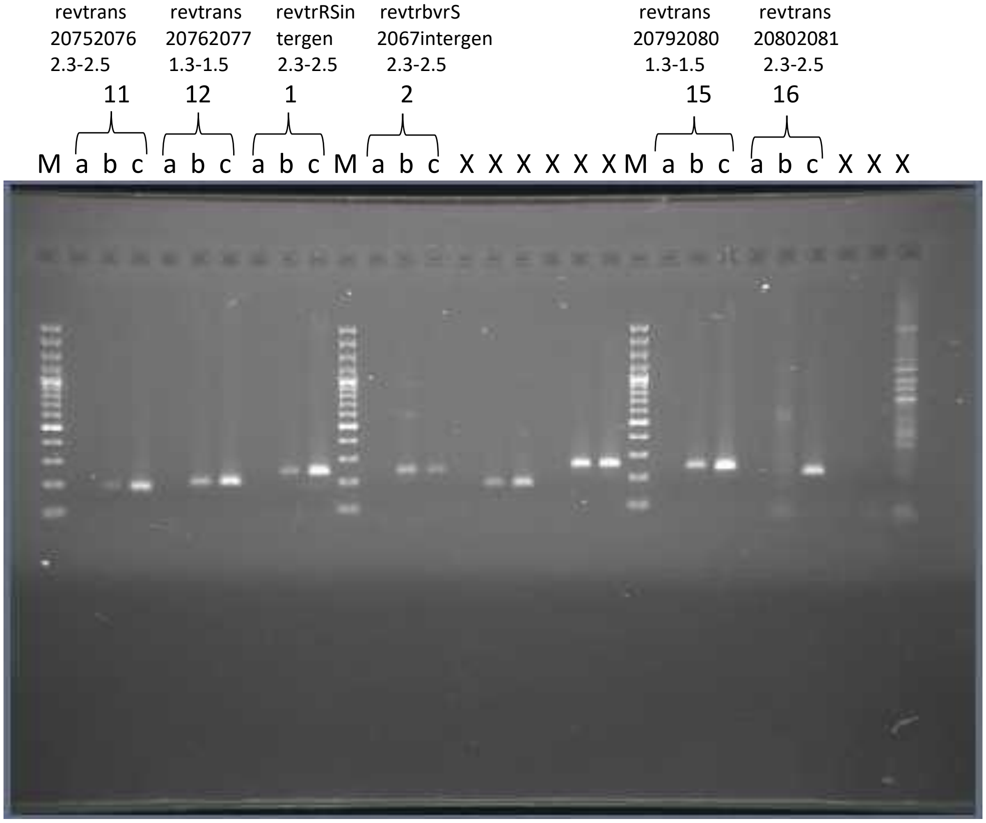

Fig 1B, samples 6,3&7, photographed gel:

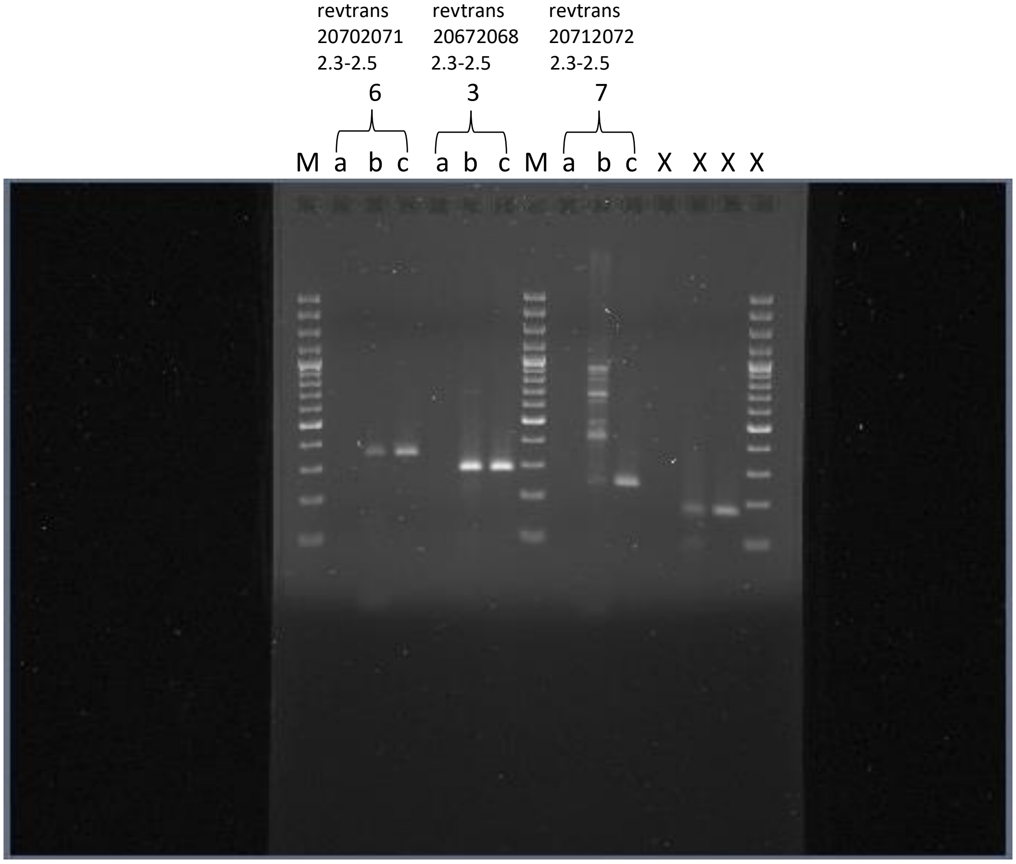

Fig 1B, samples 8,9,4&5, photographed gel:

revtrans 20722073 2.3-2.5 — 8
revtrans 20732074 .3-.5 — 9
revtrans 20682069 1.3-1.5 — 4
revtrans 20692070 1.3-1.5 — 5

M a b c a b c M a b c a b c M

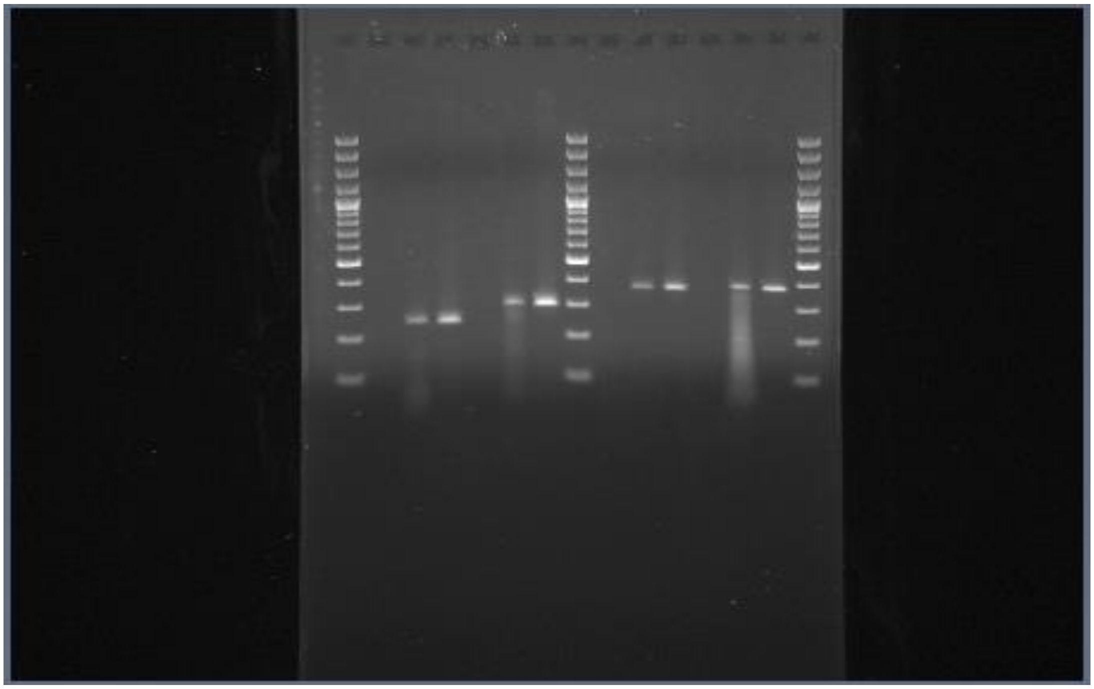

Fig 1B, sample 10, photographed gel:

revtrans
20742075
1.3-1.5
10

M a b c

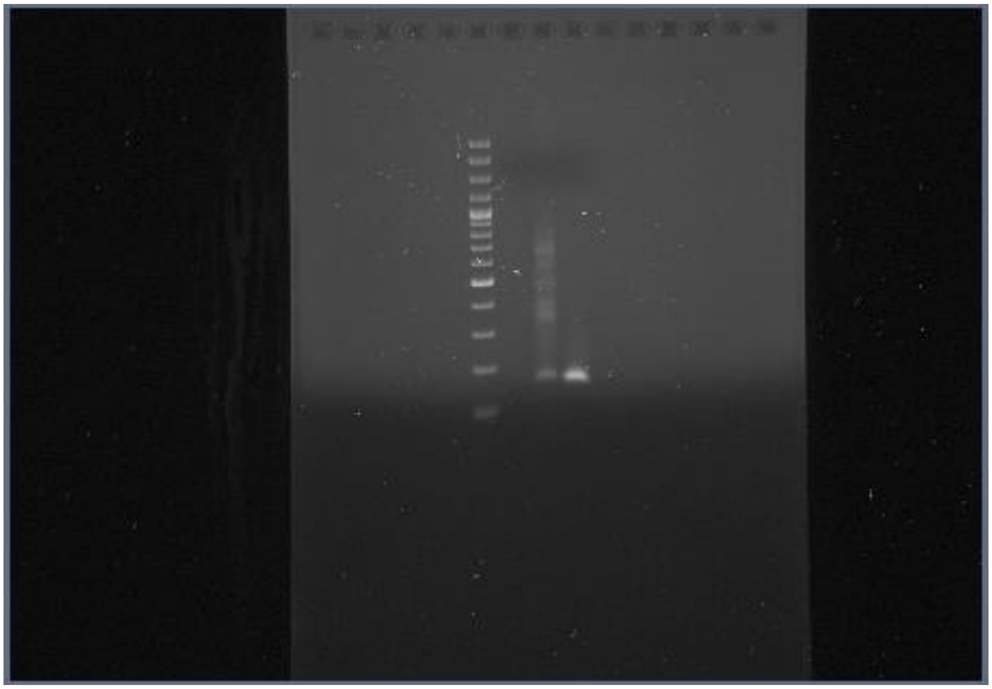

Fig 1B, samples 13&14, photographed gel:

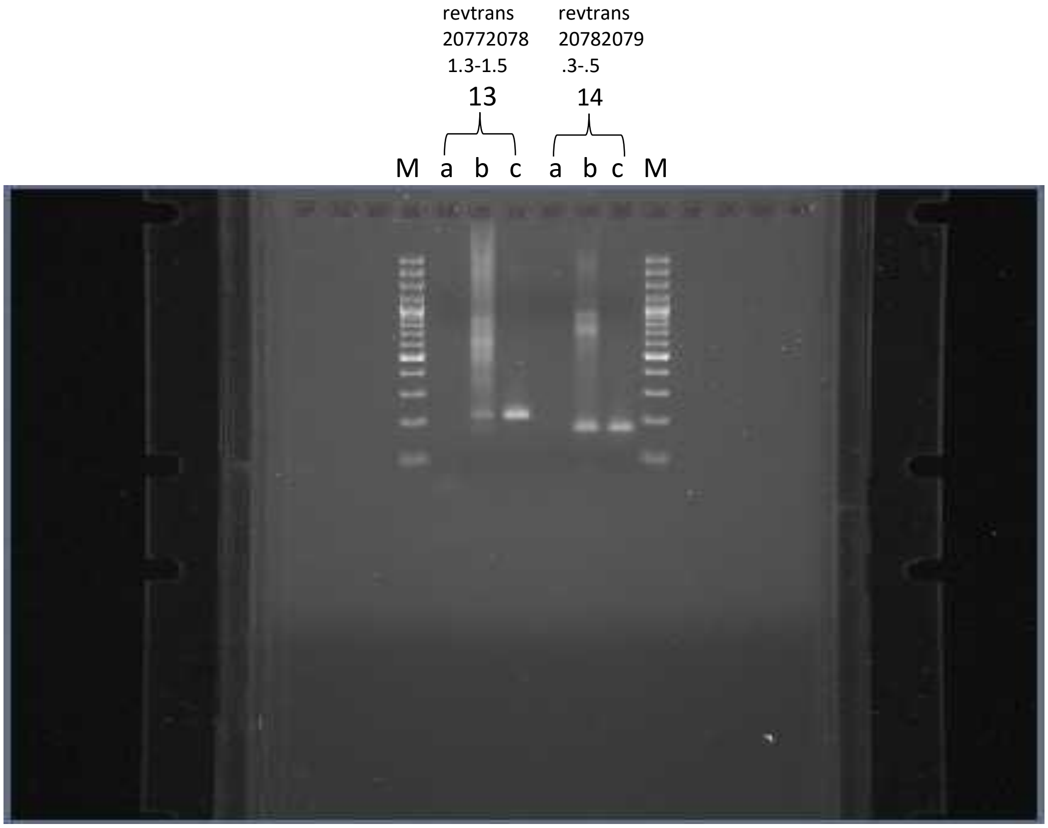

Fig 4A, gel #1, photographed film, probe *tamA*:

[BvrR] µM:	-	**0.18**	**0.37**	**0.74**	**1.11**	**1.48**	**1.85**	**2.22**	**X**	**X**

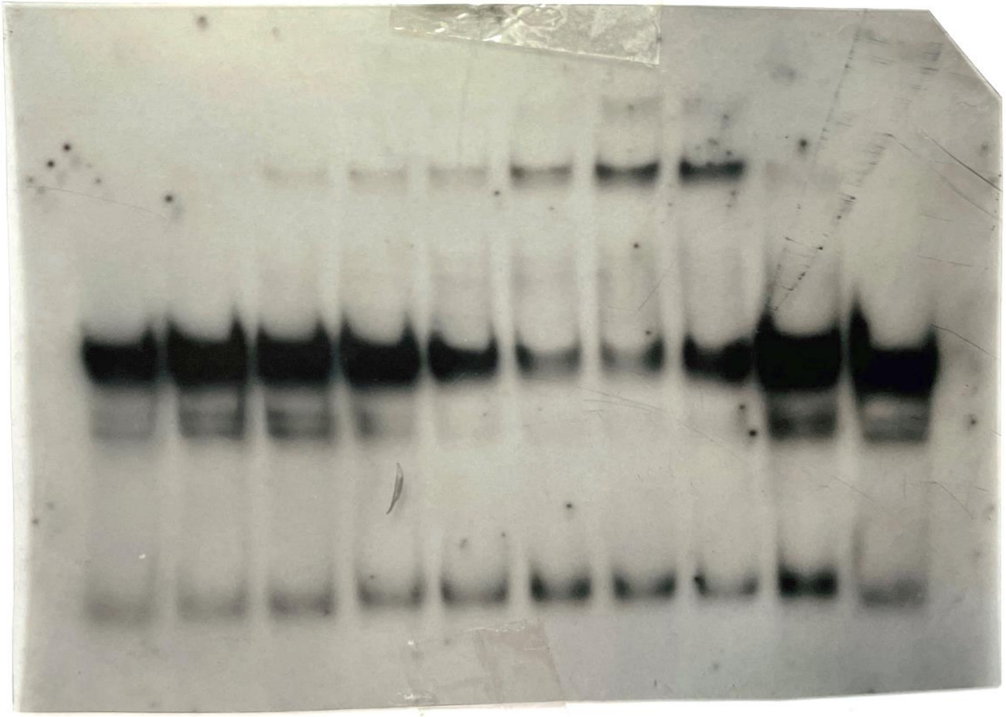

Fig 4A, gel #2, photographed film, probe *pckA*:

[BvrR] μM:	-	0.18	0.37	0.74	1.11	1.48	1.85	2.22	X

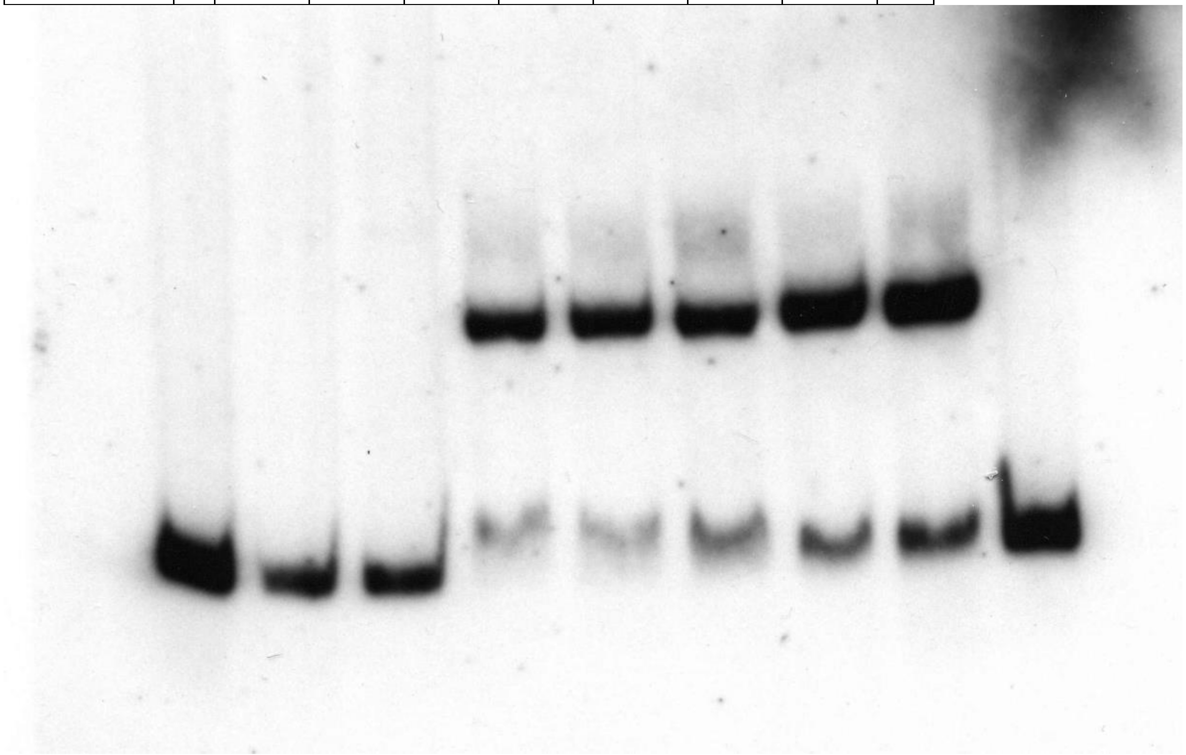

Fig 4A, gel #3, photographed film, probe *bvrR*:

[BvrR] μM:	-	0.18	0.37	0.7<br>4	1.11	1.48	1.8<br>5	2.22	X

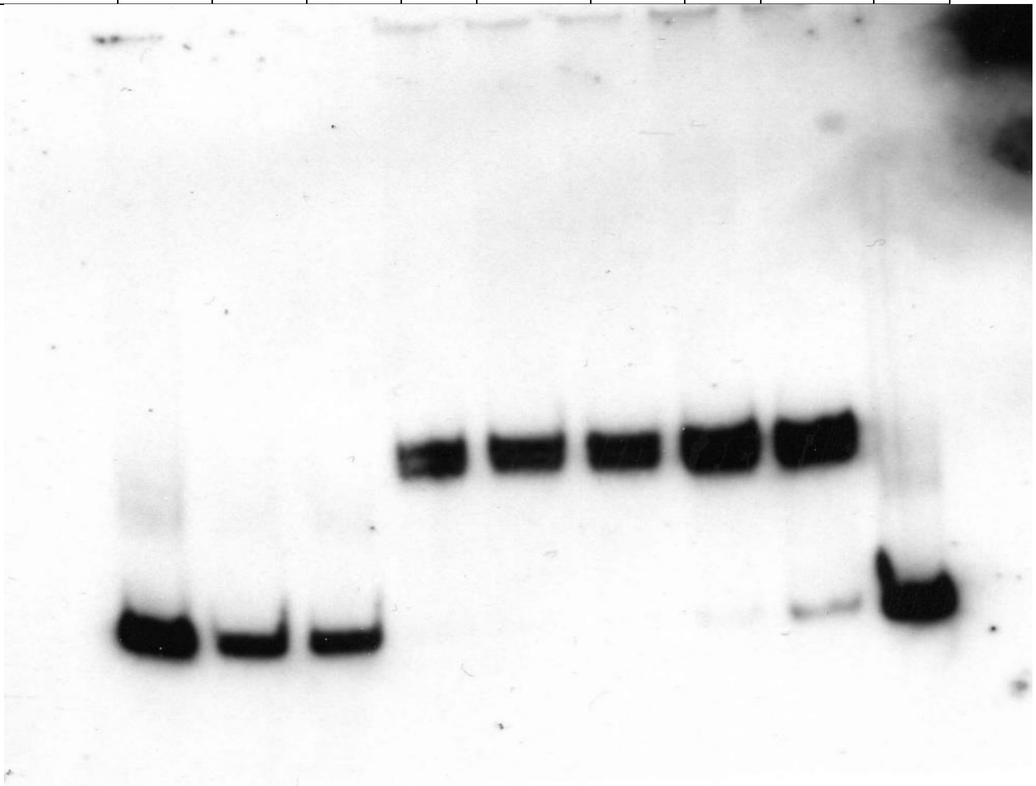

Fig 4A, gel #4, photographed film, probe *omp25*:

[BvrR] µM:	-	0.18	0.37	0.74	1.78	2.22	2.96	3.7	X	X

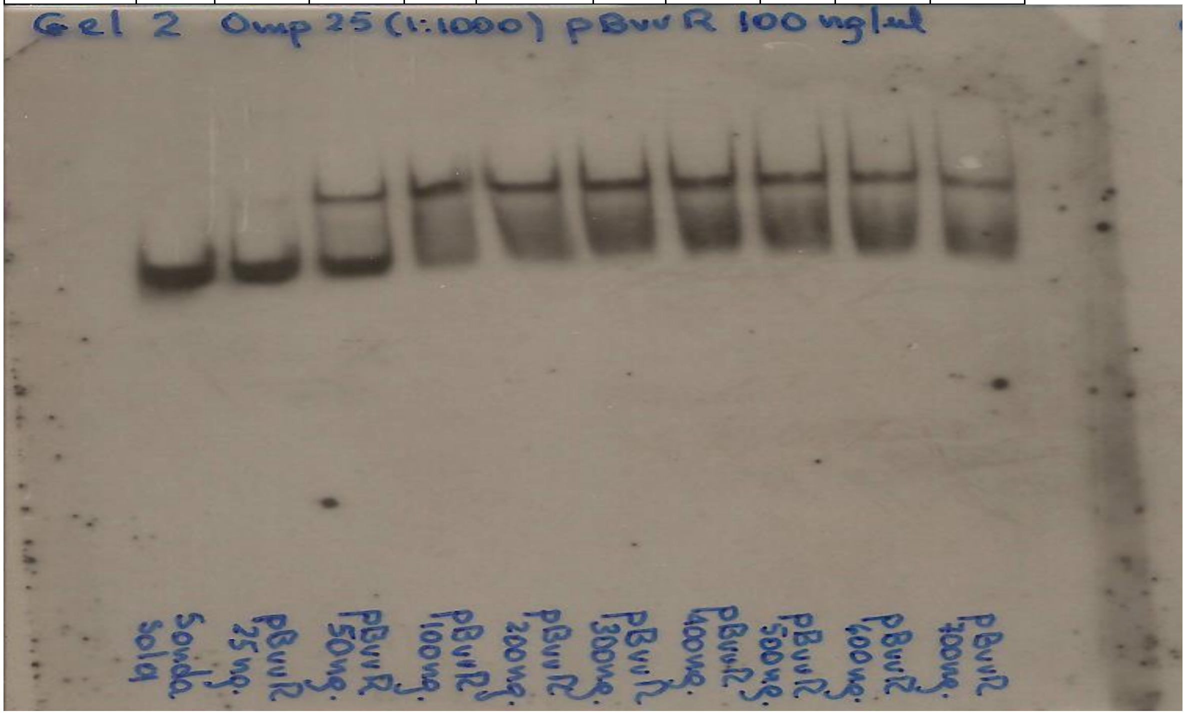

Fig 4A, gel #5, photographed film, probe *virB*:

[BvrR] µM:	-	1.11	1.48	1.85	2.22	X	X	X	X	X

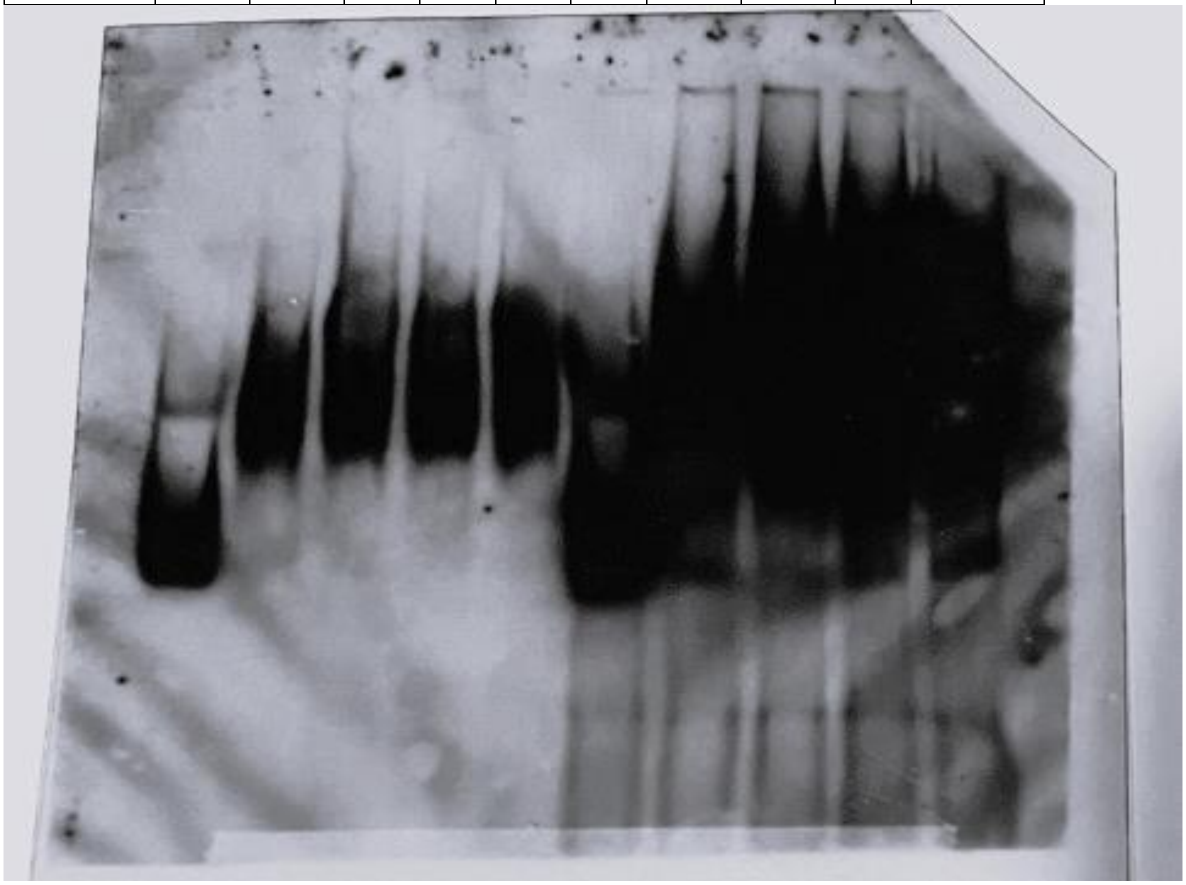

Fig 4A, gel #6, photographed film, probe *rplL*:

**[BvrR] µM:**	**X**	**X**	**-**	**0.74**	**1.11**	**1.48**	**1.85**	**2.22**	**X**	**X**

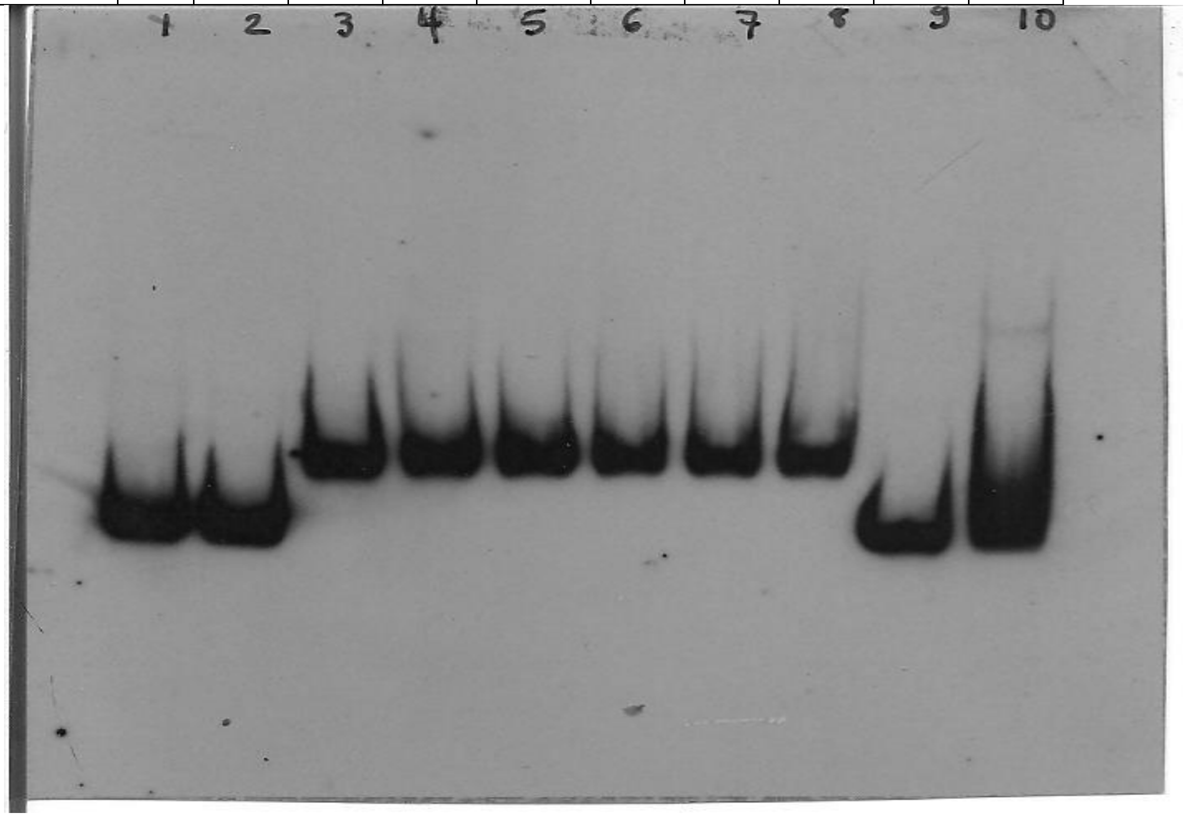

Fig 4A, gel #7, photographed film, probe *dhb*:

[BvrR] µM:	**-**	**0.18**	**0.37**	**0.55**	**0.74**	**0.92**	**1.11**	**1.48**	**X**	**X**

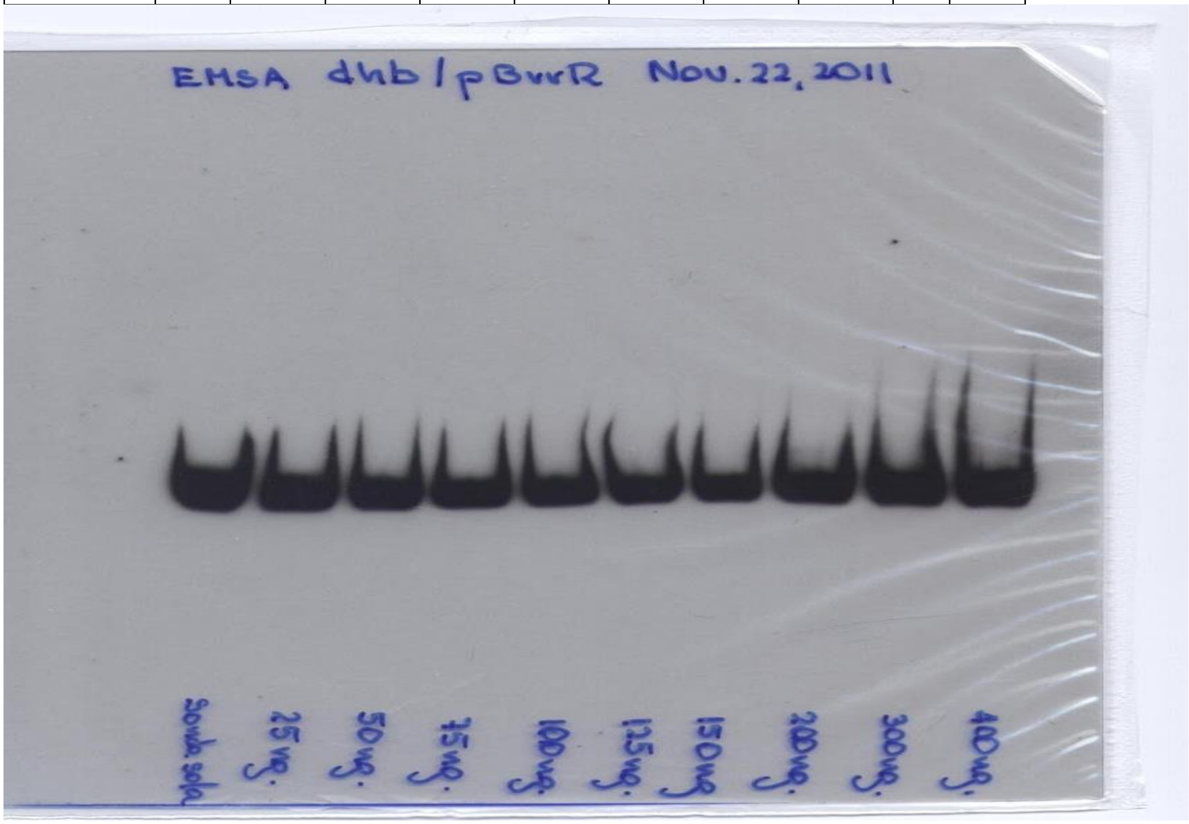

Fig 4B, gel #1, photographed film, [BvrR] = 0.6 μM, probe *tamA*, competitor *tamA*:

-	0x	250x	1000x	2000x	4000x	8000x	X	X	X

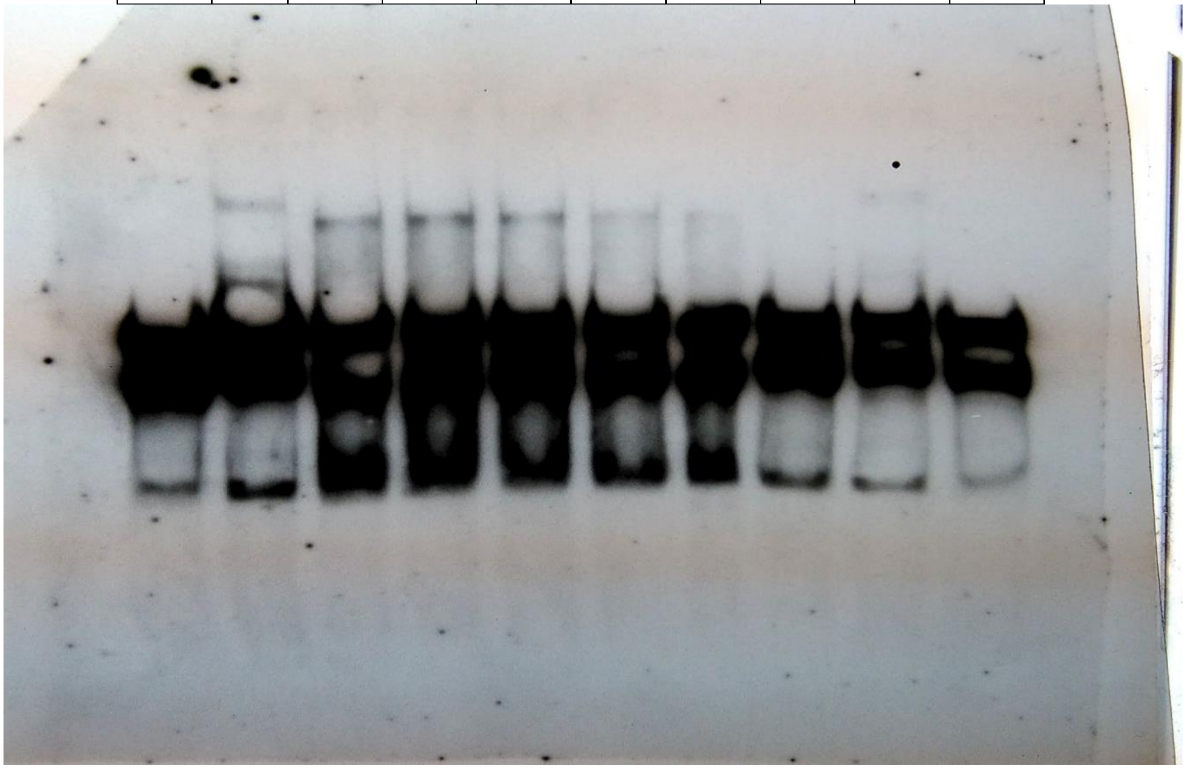

Fig 4B, gel #2, photographed film, [BvrR] = 0.6 μM, probe *tamA*, competitor *rplL*:

-	0x	250x	1000x	2000x	4000x	8000x	X	X	X

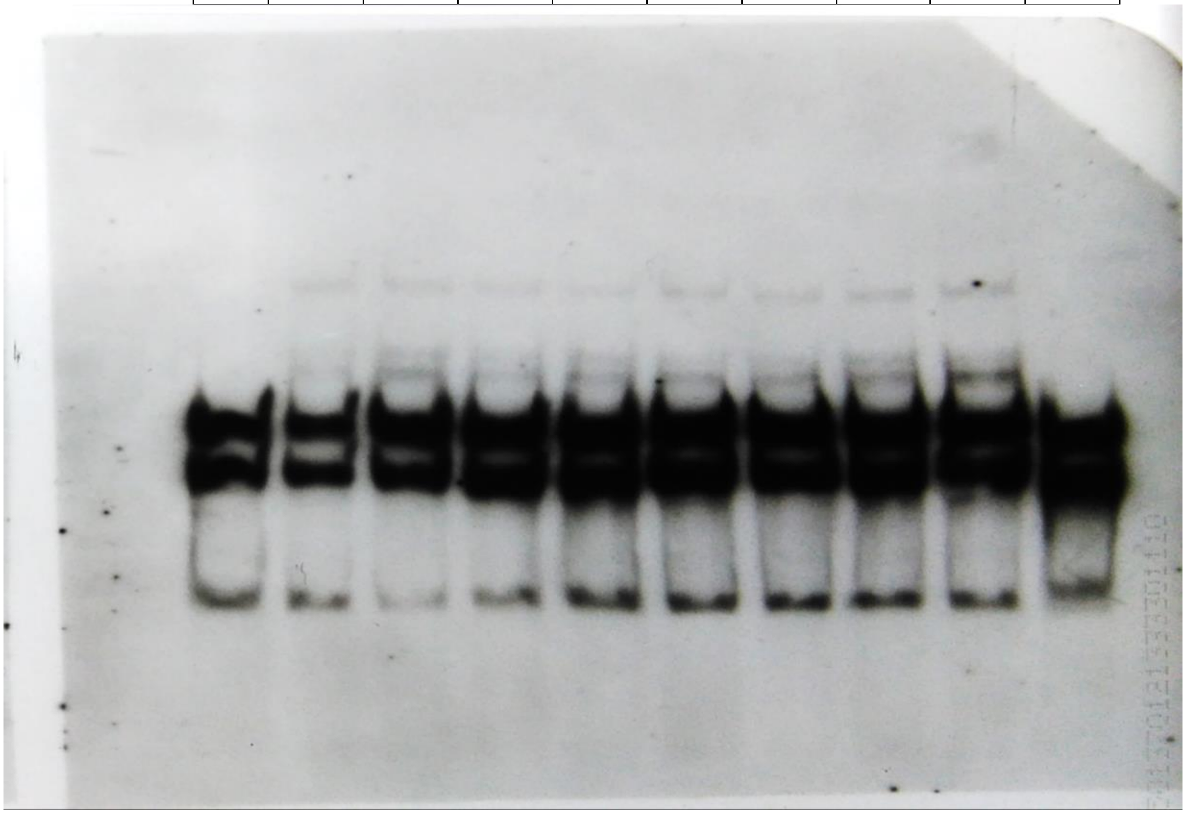

Fig 4B, gel #3, photographed film, [BvrR] = 0.6 µM, probe *omp25*, competitor *omp25*:

-	0x	8x	16x	33x	50x	66x	83x	X	X

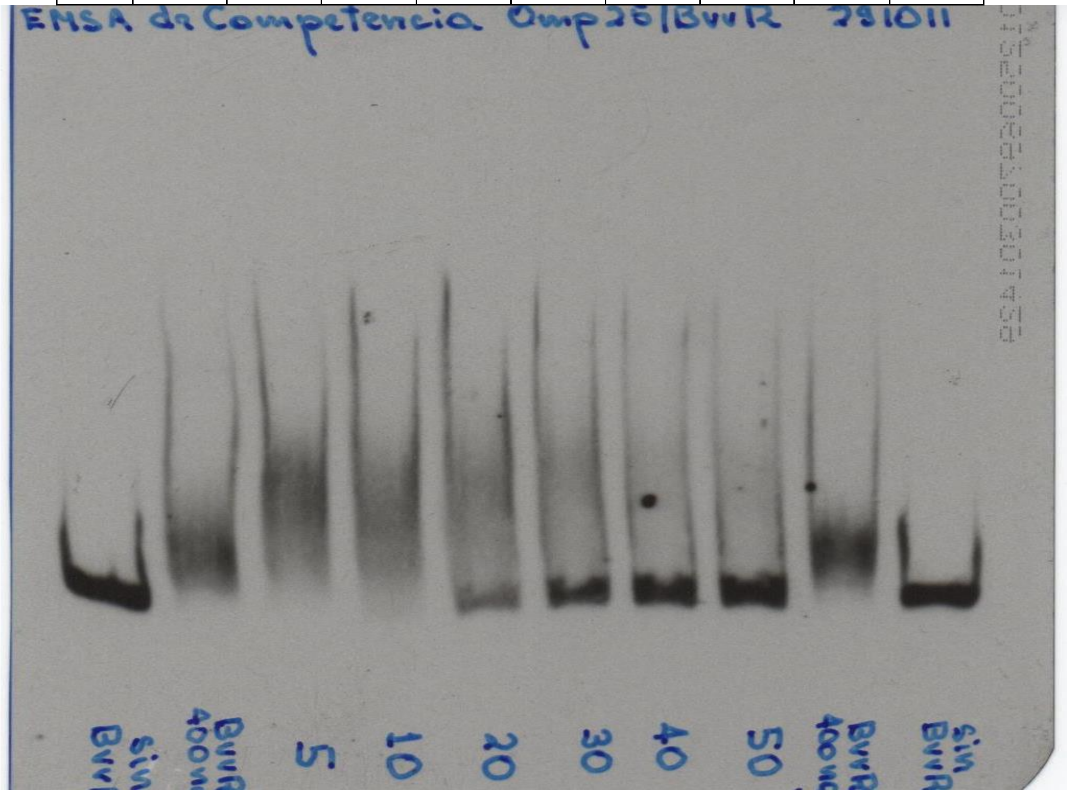

Fig 4B, gel #4, photographed film [BvrR] = 0.6 µM, probe *omp25*, competitor *dhb*:

-	0x	8x	16x	33x	50x	66x	83x	X	X

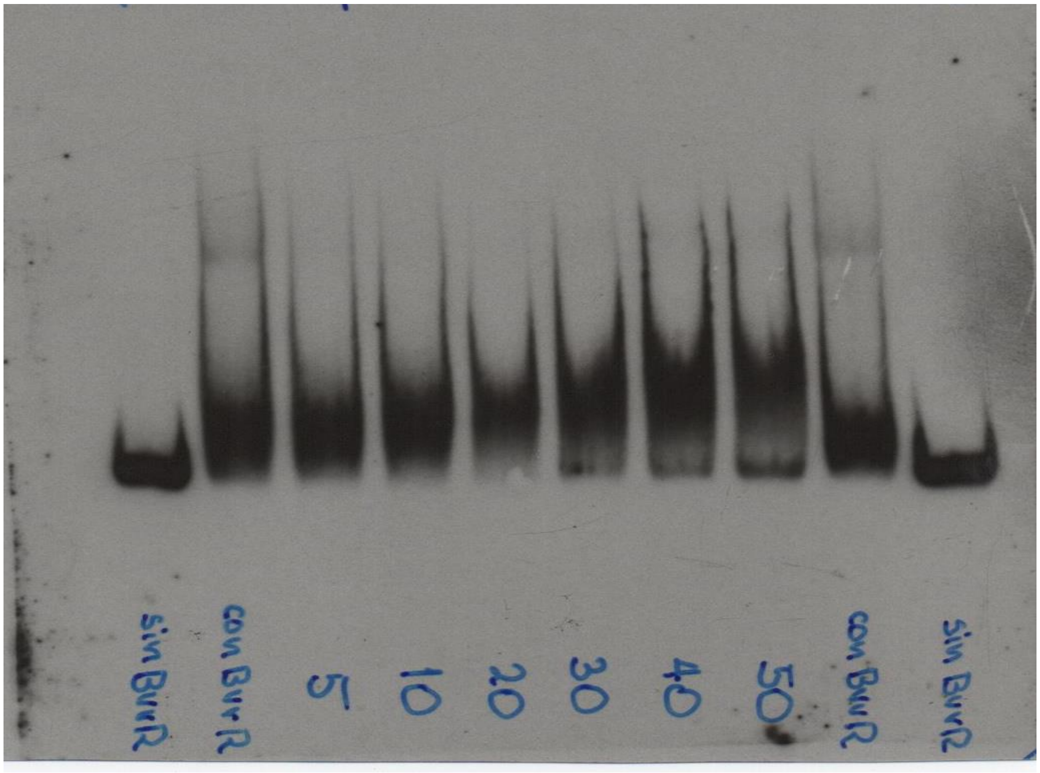

Fig 4B, gel #5, photographed film, [BvrR] = 0.6 µM, probe *virB1*, competitor *virB1*:

-	0x	25x	100x	500x	1000x	1500x	2000x	X	X

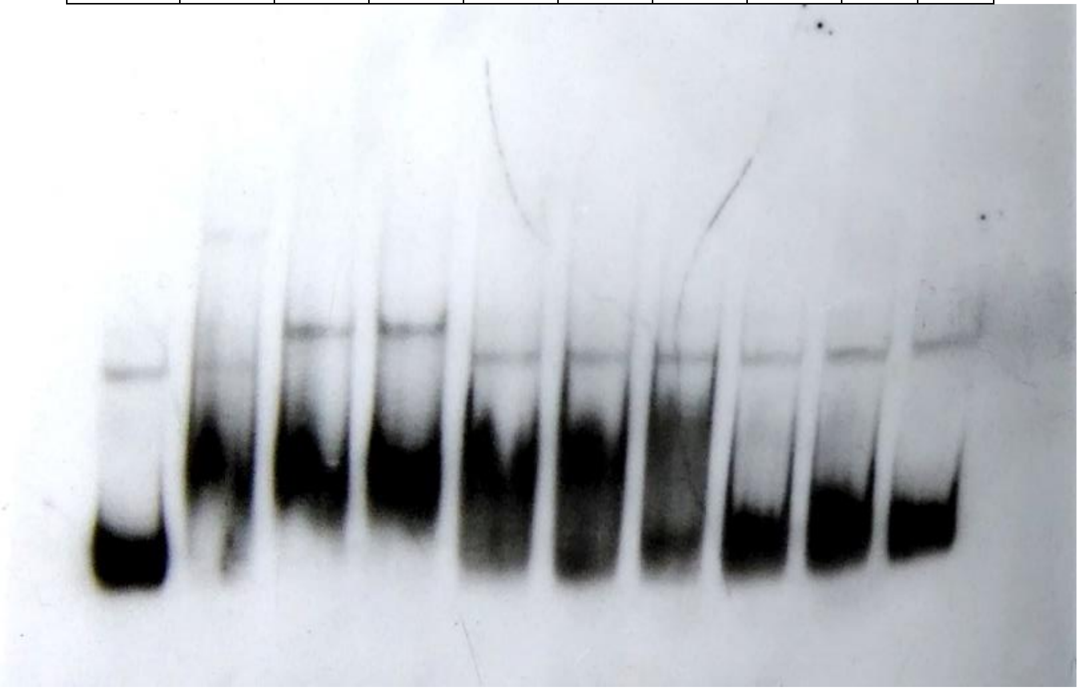

Fig 4B, gel #6, photographed film, [BvrR] = 0.6 µM, probe *virB1*, competitor *rplL*:

25x	100x	1000x	1500x	2000x	0x	-	X	X

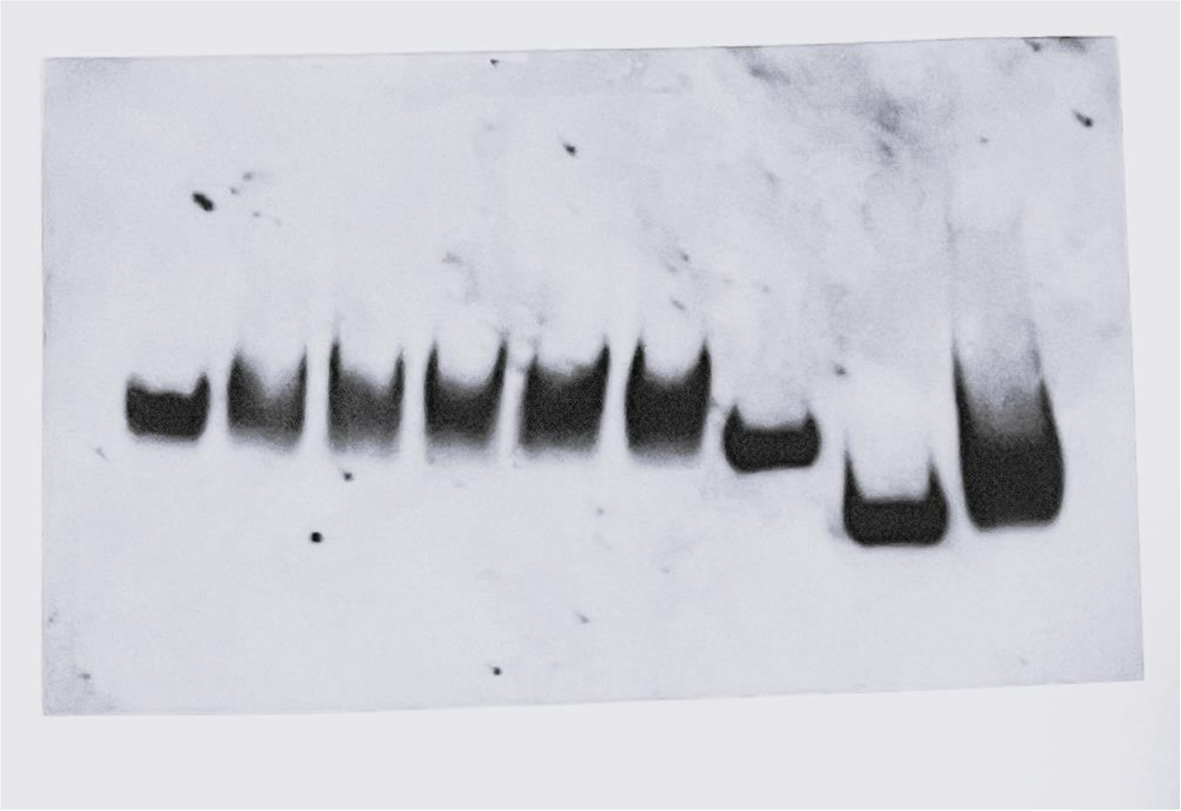

Fig 5C, gel #1, photographed film, probe *virB1* oligo 10:

[BvrR] µM:	X	0.4	0.8	1.2	1.6	2.2	2.6	-	X	X

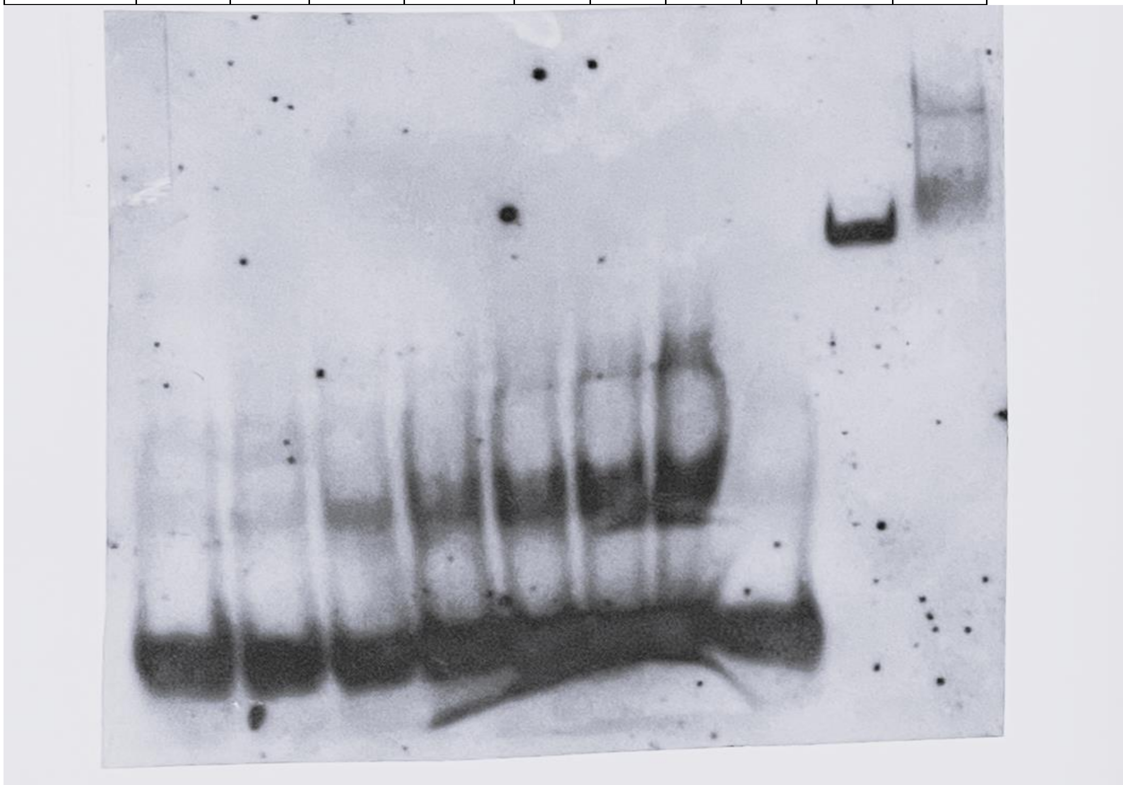

Fig 5C, gel #2, photographed film, probe *virB1* oligo 5:

[BvrR] µM:	X	0.4	0.8	1.2	1.6	2.2	2.6	-	X	X

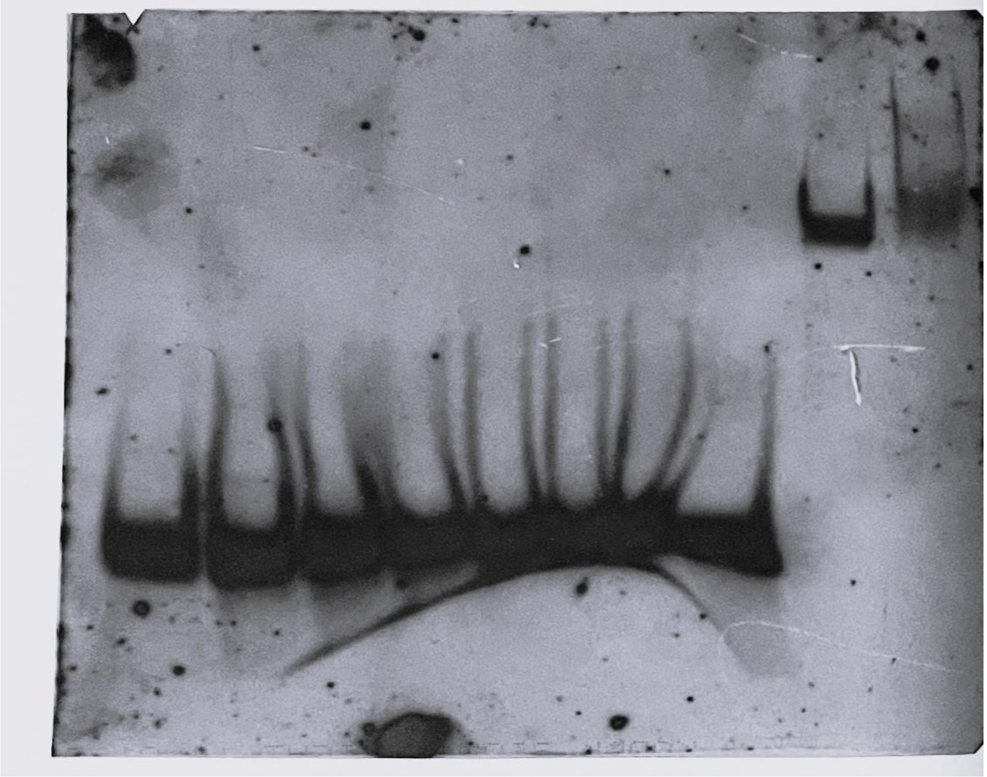

Supplement: S1 Raw images — (PDF) [file pone.0274397.s001.pdf]
