# Supplementary material for: The regulon of Brucella abortus two-component system BvrR/BvrS reveals the coordination of metabolic pathways required for intracellular life
Source: PLoS One. 2022 Sep 21;17(9):e0274397. doi: 10.1371/journal.pone.0274397 (PMC9491525; doi:10.1371/journal.pone.0274397)
Supplement: S1 Table — (PDF) [file pone.0274397.s002.pdf]

Supplementary Table 1 List of oligonucleotides used in this study.

**Name**	**Sequence (5´-3´)**
bruAb1.0048.3	CTGGATACAGGCGTTGAG
bruAb1.0048.5	CTGTTACTCCAAATCATACG
3-200pck3	GCGGCCTTATTGTGGATGC
3-200pck5	GGTAAATGTACGCCAGGGTTG
3-200bvrR3	CACTGATTCGCTTGAGGACG
3-200bvrR5	CGTCTGCGTTGCCGAAGC
omp25 For	GCCATCTTCTCGACAGATTATC
omp25 Rev	GTCTCTCGTAATCGTCTCGG
pvirdownI	GATCGTCTCCTTCTCAGAG
pvu229	CGCATACCACTTGTATATAAG
L7L12 For	GGCTGATCTCGCAAAGAT
L7L12 Rev	CCAGGTCCTTGGCTTCCTTGAG
dhbprom3	CGGCATCCAGCCATCTGCAAAA
dhbprom5	ATCACCTCACGCATGCCGCT
46.00virBO	TTAGAAAATTCTTTTTAACAAAATCTTATATACAA GTGGTATGCG
46.00virBOR	CGCATACCACTTGTATATAAGATTTTGTTAAAAAA GAATTTTCTAA
66.26virBO	GGGCACTTGTATTGGTTCTATTAGAAAATTCTTTTT TAACA

66.26virBOR	TGTTAAAAAAGAATTTTCTAATAGAACCAATACAAGTGCCC
86.46virBO	GACGCCCAGTCAGAATTTTGGGGCACTTGTATTGGTTCTAT
86.46virBOR	ATAGAACCAATACAAGTGCCCCAAAATTCTGACTGGGCGTC
106.66virBO	GGGCTATATATTGTGTGAATGACGCCCAGTCAGAATTTTGG
106.66virBOR	CCAAAATTCTGACTGGGCGTCATTCACACAATATATAGCCC
126.86virBO	GGACAAATATGCTTGTGAGGGGGCTATATATTGTGTGAATG
126.86virBOR	CATTCACACAATATATAGCCCCCTCACAAGCATATTTGTCC
146.106virBO	TGTTGTTTAAGCCGATATATGGACAAATATGCTTGTGAGGG
146.106virBOR	CCCTCACAAGCATATTTGTCCATATATCGGCTTAAACAACA
166.126virBO	TATCGGTCATTATTGGGTAGTGTTGTTTAAGCCGATATATG
166.126virBOR	CATATATCGGCTTAAACAACACTACCCAATAATGACCGATA

186.146virBO	CCTTAATTATAGATCAGCGATATCGGTCATTATTG GGTAGT
186.146virBOR	ACTACCCAATAATGACCGATATCGCTGATCTATAA TTAAGG
206.166virBO	ATGGACGATTATTATGATAGCCTTAATTATAGATC AGCGAT
206.166virBOR	ATCGCTGATCTATAATTAAGGCTATCATAATAATC GTCCAT
226.186virBO	GATCGTCTCCTTCTCAGAGAATGGACGATTATTAT GATAGC
226.186virBOR	GCTATCATAATAATCGTCCATTCTCTGAGAAGGAG ACGATC
revtrRSintergen1.3	CGCACCATCGACAGCCACATC
revtrRSintergen1.5	CGTTGCCGCCGTGCTCTG
revtransRSintergen2.3	GCTCTACGGCGTCGGTTATCG
revtransRSintergen2.5	CAAGGGCGACAGAAAGCGGC
revtrbvrS2067intergen1.3	CATTCCTATCGAGAATATCGAGC
revtrbvrS2067intergen1.5	GTGGTGGCGTGAAGACCG
revtrbvrS2067intergen2.3	GTGCCCGCTTTATCGTTGATC
revtrbvrS2067intergen2.5	GATGCAGGAGTGTGCGATCATC
revtr206720681.3	GCCTGTTCCGCATTCCCTATCG
revtr206720681.5	GACAATTTCCAACCGCACTGTG
revtrans206720682.3	GTCGGCAAATGGTGATTCCAAT

revtrans206720682.5	CCAGAGTAGCAGATGGCACTG
revtrans206820691.3	CATCCTGAAAGGTGTTGAACG
revtrans206820691.5	CTGTTCCATATCGTCTTCAGC
revtrans206820692.3	CTGCTACTCTGGCGGGTGATGCG
revtrans206820692.5	CTGATACGAACGCTCGACAGCTTGATG
revtrans206920701.3	CCGCAGGAGAATTTCGAGACTG
revtrans206920701.5	GCATTGTCGGGATCATATTCGCC
revtrans206920702.3	GAGCGTTCGTATCAGCGGTG
revtrans206920702.5	CCCACCGTCATGCCATCCTTG
revtrans207020711.3	CAGCAAGGATGGCATGACGGTG
revtrans207020711.5	CAAATCTTTATACGTGCATATTGCCCG
revtrans207020712.3	CGCTCCAGACGCTTATCGC
revtrans207020712.5	GTGCTCCAATCCAGGGCAATTC
revtrans207120721.3	CACTGATCTGCTACTCAAAACGC
revtrans207120721.5	AGCGGCTGCGACTTGC
revtrans207120722.3	GAAAATCCGCTCCAGAACC
revtrans207120722.5a	GCACCCAGAACCTTCAGCGTC
revtrans207120722.5b	GTCCAGTATTCTTCAAGCGTCTC
revtrans20722073_1.3	AGGTCGCTCGTCTTCATCTC
revtrans20722073_1.5	GATAGCCAGACCCCGTTTC
revtrans20722073_2.3	GTCCGTTCAAGTCGGAACAC
revtrans20722073_2.5	AAAAACGCACAACGCAGAC
revtrans20732074.3	GGCCGGTAACTGTTTGAAGT

revtrans20732074.5	CCTATGCTTGCCTTTCATGG
revtrans20742075_1.3	AGCTTCGTGGAACTTCATGG
revtrans20742075_1.5	GGCTTCGAGAATCTTGATCG
revtrans20742075_2.3	GTCATTTGCCGCTTTCCAT
revtrans20742075_2.5	GCCGGAAAGGGTTATGAAAT
revtrans20752076_1.3	CCCGCTTATCTGCAACATCT
revtrans20752076_1.5	ATGGGACGCATACGCTTG
revtrans20752076_2.3	ACTCGGATTGATGCAGGAAG
revtrans20752076_2.5	TGCACATTGACGATTGCAC
revtrans20762077_1.3	TCAATTGCCATTTCGATCAG
revtrans20762077_1.5	CCTCAATAAGGGCCTTTGC
revtrans20762077_2.3	GGAACACCGGAAGCCATC
revtrans20762077_2.5	ACATAGATCGTCGCGCTTG
revtrans20772078_1.3	TTCCGTGAAACCGACCTTAC
revtrans20772078_1.5	GGTCAGAACATGGGTCTTGC
revtrans20772078_2.3	CCGGAGAAGGGCTATCTTTC
revtrans20772078_2.5	CTGCGCATCGATGGTTTC
revtrans20782079.3	GCGCTGAAGGATACTCAAGG
revtrans20782079.5	TTTAACAGAATCGCCGGAAC
revtrans20792080_1.3	GAGCGGATAGAGCATTTTCG
revtrans20792080_1.5	CATTCTGCCCAGAAATCCAC
revtrans20792080_2.3	TGGAAAATGCGACAAAACAA
revtrans20792080_2.5	GGAGCGATCGTCTTGCAT

revtrans20802081_1.3	CGACGCTTCTGATGTTCAAG
revtrans20802081_1.5	TTGGGATGCAACTGCATAAG
revtrans20802081_2.3	GAGCTTGCCGCCAATATG
revtrans20802081_2.5	GATTGCCGAGCTTTTCCAG
exFAM-omp25	CGTCGGCAGCAAAAGCGGTCGCAGAGAACG
exFAM 2-omp25	CAGCTATACTGGGGAGCTACTTCAACC
exFAM2bvrR	CGCAGAAGCGCCGTCGGTATAGGTTTCG
exFAM-omp25b	GCCTTTGCGCCAGTCGCAGCAACGAGAACG
exFAM-omp3b	GGTCCCTCCCATCATGTCGGCTGCGAAAGC
exFAM-exoR	GGCAAGTGCCAAGGTAAGGACCAAGCCTAG
