# Supplementary material for: The regulon of Brucella abortus two-component system BvrR/BvrS reveals the coordination of metabolic pathways required for intracellular life
Source: PLoS One. 2022 Sep 21;17(9):e0274397. doi: 10.1371/journal.pone.0274397 (PMC9491525; doi:10.1371/journal.pone.0274397)
Supplement: S2 Table — (PDF) [file pone.0274397.s003.pdf]

Supplementary Table 2. Genomes used for α-*Proteobacteria* phylogenetic reconstruction.

Strain	Taxonomic Subgroup	Accession number
**OUTGROUP**		
Escherichia coli K12	Gammaproteobacteria	NC_000913
Geobacter sulfurreducens PCA	Betaproteobacteria	NC_002939
Ralstonia solanacearum GMI1000	Deltaproteobacteria	NC_003295
**RHIZOBIALES**		
Brucella abortus 2308 Wisconsin	Brucellaceae	GCA_900095085.1
Brucella suis str. 1330	Brucellaceae	GCA_000007505.1
Ochrobactrum intermedium LMG 3301	Brucellaceae	GCA_000182645.1
Agrobacterium tumefaciens str. Ach 5	Rhizobiaceae	NZ_CP011246.1
Sinorhizobium meliloti str. RU11/01	Rhizobiaceae	NZ_CP021219.1
Bradyrhizobium japonicum str. J5	Bradyrhizobiaceae	NZ_CP017637.1
Rodhopseudomonas palustris TIE-1	Bradyrhizobiaceae	NC_011004.1
Mesorhizobium loti str. TONO	Phyllobacteriaceae	NZ_AP017605.1
Bartonella quintana str. Toulouse	Bartonellaceae	NC_005955.1
Bartonella henselae str. BM 1374165	Bartonellaceae	NZ_HG969191.1
**CAULOBACTERALES**		
Caulobacter crescentus NA1000	Caulobacteraceae	NC_011916.1
**RICKETTSIALES**		
Rickettsia prowazeki str. RpGvF24	Rickettsiaceae	NC_017057.1
Rickettsia conorii str. Malish 7	Rickettsiaceae	NC_003103.1

Wolbachia pipientis str. wPpe	Anaplasmataceae	GCA_001752665.1
