# Supplementary material for: The regulon of Brucella abortus two-component system BvrR/BvrS reveals the coordination of metabolic pathways required for intracellular life
Source: PLoS One. 2022 Sep 21;17(9):e0274397. doi: 10.1371/journal.pone.0274397 (PMC9491525; doi:10.1371/journal.pone.0274397)
Supplement: Attachment — Submitted filename: Response to reviewer.pdf [file pone.0274397.s007.pdf]

PROGRAMA DE INVESTIGACIÓN
EN ENFERMEDADES TROPICALES
Escuela de Medicina Veterinaria, Universidad Nacional,
Apartado Postal 304-3000, Heredia COSTA RICA
Fax: 506/22381298, Tel:506/22380761,
E-mail: catguz@una.cr

San José, July the 27th, 2022

Dear Editor:

Enclosed you will find a modified version of the manuscript ID: PONE-D-22-09403: "The regulon of *Brucella abortus* two-component system BvrR/BvrS reveals the coordination of metabolic pathways required for intracellular life", in a clean and tracked changes formats.

We looked carefully into the reviewers' comments and realized that more contextualization regarding some of the findings related to the non-canonical response regulators binding sites was need it. We have introduced such contextualization in the discussion section and for length's sake we are referring the reader to literature related to this topic, which is certainly just being noticed in recent years. All comments were very helpful to improve the manuscript and considered. Figures were improved and figure captions modified accordingly. Additionally, a mistake in table 1 related to the *bvrR* locus tag was corrected, we improved Fig 5 legend and included minor drafting changes, all indicated in the track changes file. Below you will find a table answering each of the reviewers' comments, including the coordinates where they can be found in the revised version.

Thank you for your consideration. Sincerely:

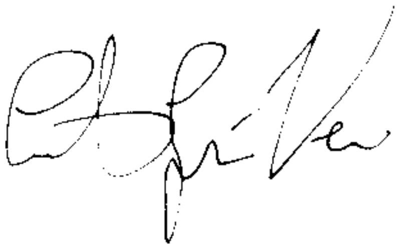

Caterina Guzmán Verri, PhD

Corresponding author

Responses to reviewer´s comments

Comment	Response
**Reviewer #1:** The manuscript by Rivas-Solano and colleagues describes the regulatory link between the BvrR/BvrS two-component system and metabolism in Brucella abortus. The authors have a long history with this system, including the identification of BvrR/BvrS and the genetic and biochemical characterization of the system, and this manuscript expands on that work to demonstrate the role that BvrR/BvrS plays in controlling the expression of genes related to metabolism. The bvrR/bvrS genes are encoded as part of a 16-gene operon, which includes may genes that putatively encode proteins involved in nitrogen metabolism, DNA repair, stress responses, and cell cycle processes. ChIP-seq analysis demonstrated that BvrR binds to more than 300 sites in the genome of B. abortus, and EMSAs confirmed direct binding to several of the identified regions. Further bioinformatic and biochemical (i.e., DNase footprinting) analyses defined a consensus DNA-binding sequence for BvrR, and the authors have developed a model for BvrR/BvrS-	*R/ We thank the reviewer for the comment.*

mediated control of metabolic systems (as well as other important virulence-related systems) in B. abortus.<br>Overall, the authors have performed a robust analysis of BvrR binding to DNA elements in B. abortus, and while many of the conclusions are supported by the data, there are some concerns about the data and conclusions.	
The specific concerns are:<br><br>-The authors have previously demonstrated that the BvrR/BvrS system is a transcriptional activator of virB. Here, the authors suggest that BvrR binds to the virB promoter at approximately -12 from the transcriptional start site. Mechanistically, this is difficult to understand. How does binding to that site promote transcription?<br><br>In the same vein, in lines 558-568 the authors discuss the different potential binding sites for BvrR, but the data in Fig. 5 using limited DNA segments show that only one region is bound by BvrR. Is it possible that multiple binding sites are needed for optimal binding?	R/ *Indeed, a BvrR, binding site on the virB promoter was found near the position -12 from the transcriptional start site. Moreover, the ChIP-seq results demonstrate potential binding sites near virB4 and virB5.*<br>*The impact of each of the described binding sites on virB transcription, as well as the existence of internal promoters in the virB region certainly deserve further consideration and is out of the scope of the presented work.*<br><br>*To our knowledge, transcription could indeed be promoted from unusual sites and multiple binding sites could be needed for optimal binding. Some activators are known to bind to unusual regions and induce a promoter activity. Since these questions are similar to question #2 from Reviewer #2, we added a line in the results section to*

introduce the idea that transcriptional regulation is a complex process of which we know very little, as follows:

Lines 461-465:

"These observations suggest that regulation of genes important for virulence is complex, and that bacterial transcription factors do not behave as per the textbook operon model with interactions between the different BvrR-P binding sites probably according to BvrR-P concentration in a given moment, and in relation to additional transcription factors that might be involved in this process, as has been described for *virB* (see below)."

This idea is described in detail in the discussion section as follows:

Lines 677-698:

"To our knowledge, transcription could indeed be promoted from unusual sites and multiple binding sites could be needed for optimal binding. Some activators are known to bind to unusual regions and induce promoter activity. as it has been described for other bacterial pathogens [100,101]. PhoP of *B. subtilis*, is a response regulator for phosphate starvation, which induces activation of *pstS* by binding to an upstream region (-40 to -132) and a coding region (+17 to +270) required for complete promoter activity. In addition, the coding region box had a low affinity for PhoP-P,

	suggesting a dynamic DNA-protein binding, in which the regulator is required to start transcription [102]. Global regulators are known to bind to a collection of sites, and the regulatory effect on each binding site would be dependent on the protein concentration at any given moment, its affinity, and in relation to additional transcription factors. Hence, they can be activators, repressors, have dual regulatory roles or no described regulatory function [103–107].<br>In *Salmonella*, the global response regulator OmpR activates the expression of SsrAB, a two-component system located on the pathogenicity island 2 (SPI-2). Several OmpR binding sites were found upstream of *ssrA*, upstream and within the *ssrB* coding sequence [108–110].<br>The BvrR binding sites described in this work should be considered *bona-fide* putative gene regulation sites that deserve further investigation. Additionally, to our knowledge, very few *Brucella* promoter regions have been functionally characterized and hence, this essential information to properly unveil the mechanisms of gene regulation is missing. In this sense, confirmation of the role of each BvrR-P binding site, by itself, or in combination with other BvrR-P binding sites and/or additional regulatory mechanisms as well as gene promoter characterization certainly will shed some light to understand this complex phenomenon.”
-Fig. 1 - This is a very minor point, but it is difficult to determine which gels/lanes correspond to the map and primer sets. Moreover, the authors have included a control to demonstrate that transcription	*R/ We thank the reviewer for this comment. Fig.1 has been revised to clarify the reviewer´s concerns. We reconstructed this figure, the regions interrogated have*

stops that the 3' end of the message (i.e., the primer set represented by the black bar). Why is a similar control not included on the 5'-end of the message?	*been re-numbered to 16 regions, and the gels have been labelled accordingly. We think this time the info is clearer. Moreover, as suggested by reviewer 2, the genes in the map have been color-coded according to their annotated function. Therefore, the colors of the genes in Fig. 2, have also been modified to match Fig.1.*<br><br>*The figure legend has been modified to clarify that the intergenic region between the 5'-gene pckA and bvrR has not been tested because it was already known that pckA transcribed independently from bvrR, bvrS and the PTS genes (Dozot et al., 2010), unlike the case of the 3´-gene folC whose relationship with the transcription of bvrR, bvrS and the PTS genes was unknown.The last primer set interrogated was not a control per se, because we did not previously know if it was co-transcribed with bvrR, bvrS and the PTS genes or not.*<br>*Each primer set was tested with a negative (RNA, no RT) and a positive control (gDNA) to compare with.*<br><br>*The revised figure legend stands as follows (lines 290-310):*<br><br>**“Fig 1. Transcriptional organization of the *bvrR/bvrS* operon in *B. abortus* 2308W.** A. Schematic representation of the genomic region encoding the *bvrR/bvrS* operon (approximate coordinates in *B. abortus* 2308W genome: 2009267-2030918). The 5´-gene *pckA* was known to transcribe independently from *bvrR*, *bvrS* and the PTS genes, unlike the 3´-genes BAW_12014 to *folC* [21]. The

	arrows indicate the orientation of transcription. The genes are color-coded according to their annotated general function: Brown = Pseudogenes and partial genes (remnants), Light blue = Regulators, Light green = Unknown, Dark green = Surface (inner membrane, outer membrane, secreted, surface structures), Yellow = Central/intermediary/miscellaneous metabolism, Red= Information transfer (transcription/translation + DNA/RNA modification). The lines below the genes illustrate the intergenic regions interrogated with primer pairs listed in Table S1, and are numbered from 1 to 16, according to their intergenic position along the operon. Black = co-transcribed regions as demonstrated by RT-PCR, Gray = non-co-transcribed region as demonstrated by RT-PCR. B. Agarose gel electrophoresis of RT-PCR products obtained per region interrogated. For each RT-PCR result numbered from 1 to 16, three lanes are shown: a-minus RT (RNA, no RT), b-RT-PCR result and c-positive control (gDNA). The last 5 bands of the molecular marker (M) are 100, 200, 300, 400 and 500 bp-long. In total, 31 primer pairs were tested to span 16 overlapping regions of no more than 400bp. Only one representative RT-PCR product per region is shown. All amplicons were sequenced to corroborate their identity. The results shown correspond to the log phase of the growth curve in TSB and are also representative of the co-transcription events observed at the stationary growth phase in the same medium.”
-Fig. 4 - Overall, the EMSAs are convincing, but there are some issues with some of them. For example, the binding to the virB	*R/The experiments shown in panels A and B are different and independent from each other (direct EMSA and*

promoter is highly variable between panels A and B. Why is this? It is understandable that differences exists between experiments, but in this case, the data are very difficult to interpret in terms of the competition controls when the control for those experiments looks nothing like the results in the panel A.	*competitive EMSA respectively). Therefore, they are not meant to be compared in between. Each gel, either on panel A or B, has its own negative control (probe without protein) to compared with. In panel B, each gel has its own positive control (probe with protein) to compare with.*<br>*In the case of the virB probe:*<br>*-The direct EMSA shown in panel A has its negative control on lane 1. Thus, the migration pattern of the probe incubated with growing concentrations of the protein (lanes 2-4) must be compared against lane 1. As shown, there is indeed a difference in the migration pattern (shift) observed in lanes 2-4, when compared to lane 1, which indicates a molecular interaction between BvrR and the virB probe.*<br>*-The competitive EMSA shown in panel B has its negative control on lane 1 and its positive control on lane 2. Thus, the migration pattern of the probe incubated with the same concentration of protein as the positive control and with growing concentrations of the competitor (excess of "cold", ie non-labelled virB probe) should be compared against lanes 1 and 2. As shown, there is a shift in the positive control, and this shift is progressively reduced as the concentration of the cold virB probe competitor increases, which indicates a specific molecular interaction between BvrR and the cold virB probe. In the case of the competition experiment between the virB probe and the "cold" rplL probe, the negative control is in the last lane, and the positive control in the lane next to that. Indeed, this positive control shows a somewhat different migration pattern as compared to the virB positive controls in panel A and in the gel with virB cold probe. In our experience of more than five years running EMSAs, the pattern shift*

	obtained is difficult to reproduce exactly the same in each experiment. We think this is due to the fact that salt concentration and BvrR phosphorylation are factors difficult to control and affect DNA-protein interaction, DNA and protein conformation. Hence the importance of always using controls in each run experiment to compare to.<br>*All the gels shown in figure 4 should be evaluated the same way as explained for the virB probe.*<br><br>*To clarify this, the following sentences have been added to the revised figure legend (lines 486-489):*<br><br>"Experiments in panels A and B are independent from each other. All gels have either negative (probe without protein) and/or positive controls (probe with protein) to compare with."<br><br>*Additionally, a more detailed explanation on how the competitive EMSAs are performed was introduced besides the conventional EMSA as follows (Lines 259-263):*<br><br>"Competitive EMSA were performed as described (10). Briefly, the digoxigenin-labeled probes tested in the direct EMSA for *tamA*, *omp25* and *virB1*, were incubated with BvrR-P (0.6 µM) and either an excess of the respective non-labeled probe as competitor, or separately, with an excess of non-labeled negative control probe (*rplL* or *dhb*) as competitor. Samples were then processed as described for direct EMSAs.

-Line 68 - Brucella replicates in a vacuole composed of (or associated with) the ER, and thus it may be incorrect to say that the bacteria replicate "within the ER."	R/*The sentence has been modified according to the reviewer's suggestion and a reference has been added:*<br>*Lines 68-70:*<br>"After two days of intracellular life, bacteria extensively replicate in a vacuole associated with the ER and restore to pre-infection levels most of the differentially expressed proteins [5]".
**Reviewer #2:** This study aims to expand our knowledge of the genes controlled by the Brucella BvrR/S two component system.<br>While the experiments are well performed and the data presented solid, the conclusions are not fully supported by the data, more experimental work is needed.	R/*We agree with the reviewer comment in the sense that BvrR-P binding to DNA in vitro conditions is not in itself demonstration of gene regulation. Indeed, more experimental work is need it to stablish if this is the case of each of the putative target genes. The evidence presented in this manuscript suggests that BvrR/BvrS gene regulation is a complex process that at DNA level probably involves binding of BvrR-P molecules in more than one single site and in non-canonical E. coli regions. Additionally, to our knowledge, very few Brucella promoter regions have been functionally characterized and hence this essential information to properly unveil the mechanisms of gene regulation is missing. In this sense, confirmation of the role of each BvrR-P binding site, by itself, or in combination with other BvrR-P binding sites and/or additional regulatory mechanisms as well as gene promoter characterization certainly will shed some light to understand this complex phenomenon, which is out of the scope of this manuscript. We are preparing an entire new manuscript related to the characterization of the omp25 regulation exerted by BvrR/BvrS and the impact that each found binding site has on its transcription.*<br>*We modified part of the results section to include the idea that more research is need it to establish the role of the found BvrR binding sites:*

	Lines 426-432<br><br>"As anticipated, BvrR/BvrS seems to regulate other metabolic pathways related to membrane composition and virulence (Table 2) [20,58,59]. Altogether, as expected from previous work, these results suggest that BvrR/BvrS TCS regulates crucial pathways vital for intracellular trafficking and survival. This is probably achieved by directly regulating enzymes located at crossroads or in tandem of these metabolic pathways [5,57]. More work is need it to establish if these *bona-fide* BvrR-P binding sites are indeed gene regulation sites."<br><br>*We have also modified the discussion accordingly, so the convey message is that the binding sites found should be considered bona-fide putative gene regulation sites that deserve further investigation:*<br><br>*Lines 676-697*<br><br>"To our knowledge, transcription could indeed be promoted from unusual sites and multiple binding sites could be needed for optimal binding. Some activators are known to bind to unusual regions and induce promoter activity. as it has been described for other bacterial pathogens [100,101]. PhoP of *B. subtilis*, is a response regulator for phosphate starvation, which induces activation of *pstS* by binding to an upstream region (-40 to -132) and a coding region (+17 to +270) required for complete promoter activity. In addition,

	the coding region box had a low affinity for PhoP-P, suggesting a dynamic DNA-protein binding, in which the regulator is required to start transcription [102]. Global regulators are known to bind to a collection of sites, and the regulatory effect on each binding site would be dependent on the protein concentration at any given moment, its affinity, and in relation to additional transcription factors. Hence, they can be activators, repressors, have dual regulatory roles or no described regulatory function [103–107].<br>In *Salmonella*, the global response regulator OmpR activates the expression of SsrAB, a two-component system located on the pathogenicity island 2 (SPI-2). Several OmpR binding sites were found upstream of *ssrA*, upstream and within the *ssrB* coding sequence [108–110].<br>The BvrR binding sites described in this work should be considered *bona-fide* putative gene regulation sites that deserve further investigation. Additionally, to our knowledge, very few *Brucella* promoter regions have been functionally characterized and hence, this essential information to properly unveil the mechanisms of gene regulation is missing. In this sense, confirmation of the role of each BvrR-P binding site, by itself, or in combination with other BvrR-P binding sites and/or additional regulatory mechanisms as well as gene promoter characterization certainly will shed some light to understand this complex phenomenon.”
Major Concerns<br>1 The authors write (L114) ‘We expand our knowledge of the BvrR/BvrS regulon,	*R/The sentence has been modified as follows to clarify the reviewer´s concern:*

describing the genes controlled directly by this TCS and under conditions that mimic the intracellular environment confronted by B. abortus while trafficking to its replicative niche.'<br>This is not correct, the data show binding of Bvr-P to DNA, not regulation of gene expression. While there is some evidence that BvrR/S controls expression some genes, including virB and omp25, yo support this claim, it is essential that the authors provide data for the new set of genes that they claim to be controlled by BvrR/S.	Lines 120-122:<br>"We expand our knowledge of the BvrR/BvrS regulon, describing genomic regions directly bound by BvrR under conditions that mimic the intracellular environment confronted by *B. abortus* while trafficking to its replicative niche."
2 The authors say that most BvrR-P binding sites are in regions upstream of the target genes. They also find binding sites in the virB4 and virB5 genes, several thousand bases into the operon. This is not at all discussed or commented on in the manuscript. How does this work? Are there internal promotors? This should be clarified.	R/*According to the info presented in Supplementary Table 4, most of the BvrR-P binding sites are within coding regions, as has been described in several cases (Bonocora RP, et al. 2015 PLoS Genet. Lybecker M,et al. 2014. Transcription, Fitzgerald DM, et al 2014. PLOS Genet). The fact that there are still many general questions to answer related to transcriptional regulation in prokaryotes (Mejía-Almonte C, et al. 2020. Nat Rev Genet), and even more within the Brucella genus, precludes the possibility for establishing hypothesis that could be easily proven. Information regarding promoter structure, position of transcriptional binding sites and transcriptional regulators in Brucella is scarce. As mentioned in the response to the first question, the evidence presented in this manuscript suggests that BvrR/BvrS gene regulation is a complex process that at DNA level probably involves binding of BvrR-P molecules in more than one single site and in non-canonical E. coli regions* including coding regions and/or downstream *promoter regions (Liu et al. 1998. Mol Microbiol. 119–130, Shimada T et al. 2008. The Nucleic Acids Res 36:3950.) or even in a promoter region, located in a coding region (Fitzgerald DM, et al. 2018. Mol*

	Microbiol 108:361–378.). The information presented in this manuscript is intended to present a first glance of such gene regulation process, in relation to BvrR/BvrS.<br>*Hence, the fact that binding sites found within operons, as is the case of the virB4 and virB5 opens many possibilities, from additional promoter regions to binding regions with no regulatory function at all. The answer to this question is certainly relevant, and out of the scope of this manuscript.*<br>*We have introduced three paragraphs in the discussion section to address the reviewer concern, in a general way, to keep the manuscript script (Lines 676-697*<br>*)*, and described *in the first answer to reviewer #2.*<br>*Additionally, we replaced the following sentence in the results section:*<br><br>"Furthermore, through ChIP-Seq, we detected five different binding sites for *virB1* (Table 3 and Fig 5F), suggesting that additional TSS located within the coding region could be expressed under different conditions [8,78]."<br>*For the following sentence: (lines 493 to 496)*<br>"Furthermore, through ChIP-Seq, we detected five different binding sites related to *virB* (Table 3 and Fig 5F), suggesting that additional TSS located within the coding region could be expressed under different conditions [8,79]."
3 Fig 3. Stress conditions increase binding of BvrR. Is this specific to BvrR. What happens with another TCS regulator, will it also bind to its targets more efficiently?	R/ *The increase in BvrR binding sites observed under stress conditions is not specific for BvrR. Some examples are the response regulator OmpR in E. coli and in Salmonella Typhimurium (A new role of OmpR in acid and*

	osmotic stress in Salmonella and E. coli. Frontiers in Microbiology, 2656.*Chakraborty & Kenney, 2018). This also seems to be the case of the TCS ChvGI in Caulobacter crescentus in response to cell envelope stress (The two-component system ChvGI maintains cell envelope homeostasis in Caulobacter crescentus. Alex Quintero-Yanes, Aurélie Mayard, Régis Hallez bioRxiv 2022.01.18.476748; doi: https://doi.org/10.1101/2022.01.18.476748)*<br><br>*To keep the manuscript as simpler as possible, we introduced a statement in the results section and pointed the reader to the published reference as follows:*<br><br>Line 385-388:<br>"Analysis of the function category of the closest gene to a significant signal showed that the number of genes in all functional categories detected under rich conditions increased under stress conditions (Fig 3A and S4 Table), an observation that has also been described in other pathogens' TCSs (53)."
Other concerns<br>4 Fig 1 hard to follow with respect to text, there is a confusing mix of mix of gene names and gene numbers. It would be easier to follow if the figure showed the gene names referred to in the text. A more extensive color code could also help with calrity…so all pts genes in one color, unknown function in another etc.	*R/ Fig.1 has been modified to address both reviewer´s concerns. Genes have been color-coded according to the annotated general function and therefore, the color-code of the genes in Fig. 2 have also been changed to match the same color code from Fig 1. Other changes have been introduced in Fig.1 and its legend, as suggested by the reviewer 1.*<br>*The revised legend of Fig.1 stands as follows lines 290-310:*

	"**Fig 1. Transcriptional organization of the *bvrR/bvrS* operon in *B. abortus* 2308W.** A. Schematic representation of the genomic region encoding the *bvrR/bvrS* operon (approximate coordinates in *B. abortus* 2308W genome: 2009267-2030918). The 5´-gene *pckA* was known to transcribe independently from *bvrR*, *bvrS* and the PTS genes, unlike the 3´-genes BAW_12014 to *folC* [21]. The arrows indicate the orientation of transcription. The genes are color-coded according to their annotated general function: Brown = Pseudogenes and partial genes (remnants), Light blue = Regulators, Light green = Unknown, Dark green = Surface (inner membrane, outer membrane, secreted, surface structures), Yellow = Central/intermediary/miscellaneous metabolism, Red= Information transfer (transcription/translation + DNA/RNA modification). The lines below the genes illustrate the intergenic regions interrogated with primer pairs listed in Table S1, and are numbered from 1 to 16, according to their intergenic position along the operon. Black = co-transcribed regions as demonstrated by RT-PCR, Gray = non-co-transcribed region as demonstrated by RT-PCR. B. Agarose gel electrophoresis of RT-PCR products obtained per region interrogated. For each RT-PCR result numbered from 1 to 16, three lanes are shown: a-minus RT (RNA, no RT), b-RT-PCR result and c-positive control (gDNA). The last 5 bands of the molecular marker (M) are 100, 200, 300, 400 and 500 bp-long. In total, 31 primer pairs were tested to span 16 overlapping regions of no more than 400bp. Only one

	representative RT-PCR product per region is shown. All amplicons were sequenced to corroborate their identity. The results shown correspond to the log phase of the growth curve in TSB and are also representative of the co-transcription events observed at the stationary growth phase in the same medium.”
5 The legend for Fig1B does not fit with the figure. First, the authors write that there are 31 primer pairs; this implied 31 PCR reactions, why are only 15 shown. If this is an English language problem and the authors meant 15 primer pairs (so 30 primers), where doses the number 31 come from?	R/As previously stated, Fig.1 has been modified and hence its legend. We apologize for the confusion. Indeed, there are 31 primer pairs<br><br>Fig 1B legend is now (lines 303-310):<br><br>“B. Agarose gel electrophoresis of RT-PCR products obtained per region interrogated. For each RT-PCR result numbered from 1 to 16, three lanes are shown: a-minus RT (RNA, no RT), b-RT-PCR result and c-positive control (gDNA). The last 5 bands of the molecular marker (M) are 100, 200, 300, 400 and 500 bp-long. In total, 31 primer pairs were tested to span 16 overlapping regions of no more than 400bp. Only one representative RT-PCR product per region is shown. All amplicons were sequenced to corroborate their identity. The results shown correspond to the log phase of the growth curve in TSB and are also representative of the co-transcription events observed at the stationary growth phase in the same medium.”
6 The authors write ‘These co-transcription events happened at log and stationary growth phases…’ however they do not show data for different growth phases. What was the growth phase tested in Fig 1?	*R/ Co-transcription was observed at both log and stationary growth phases, only the results of the former were chosen to construct Fig. 1, to avoid an oversized and repetitive figure. As mentioned, the legend has been*

	modified as described above to clarify the reviewer´s concern:<br>Lines 303-310,<br>and consequently removed the following sentence from the results section:, L285; "As demonstrated, co-transcription is independent of the growth stage".
7 The introduction is rather confused. Paragraph from L72-Are you talking about TCS in general or Brucella and BvrR/S? The refs suggest the latter the text the former.<br><br><br><br><br><br><br><br><br><br><br><br><br><br><br><br><br><br>L73 Define TCS	*R/ According to the reviewer´s suggestion, we changed the references related to TCS in general, as follows:*<br><br>....." The phosphorylated form of this protein shows an increased affinity for DNA binding sites, activating or repressing a particular set of genes, which constitute a direct regulon [7,8].<br>*Is now replaced for (starting at line 82):*<br><br>....." The phosphorylated form of this protein shows an increased affinity for DNA binding sites, activating or repressing a particular set of genes, which constitute a direct regulon [6].<br><br><br><br>*According to the reviewer´s suggestion, the previous definition of TCSs in L73:*<br><br>"The transition from an extracellular to an intracellular milieu requires a highly coordinated gene expression. This is achieved through several regulatory mechanisms, including TCSs that allow

	bacteria to sense and respond to environmental variations"<br><br>*It is now replaced for (starting at L76):*<br><br>"The transition from an extracellular to an intracellular milieu requires a highly coordinated gene expression. This is achieved through several regulatory mechanisms, including TCSs: *signal transduction systems* that allow bacteria to sense and respond to environmental variations [6]
Paragraph from L80.<br>Here there are mixed references to TCS then PTS.<br><br>It would be much clearer to introduce the Bvr family in the alphas and then talk about PTS.	*R/We reviewed all references in paragraph starting at previous L80 (now starting at L85). This paragraph was modified according to the next reviewer´s comment below.*<br><br>*R/ We thank the reviewer for this suggestion. The PTS system and its relation to the Bvr family is now after the description of BvrR/BvrS in the introduction section (please see moved tracked change in the introduction section, starting at line 87)*
8 L69 Bacteria then reach an autophagosome-like exit compartment where they are ready to egress from the host cell and start a new infection cycle [5]<br>Not an appropriate reference	*R/We agree with the reviewer. The reference has been changed for:*<br>Line 70:<br>Starr T, Ng TW, Wehrly TD, Knodler LA, Celli J. *Brucella* intracellular replication requires trafficking through the late endosomal/lysosomal compartment. Traffic. 2008;9(5):678–94.

9 L94 ‘Bacteria grown in a nutrient-rich medium at neutral pH (rich conditions), transiently activate BvrR through phosphorylation’<br>Modify to ‘When bacteria are grown in a nutrient-rich medium at neutral pH (rich conditions), BvrR is transiently activated through phosphorylation’	*R/The sentence has been modified as follows, according to the reviewer´s suggestion (now L90):*<br><br>“When bacteria are grown in a nutrient-rich medium at neutral pH (rich conditions), BvrR is transiently activated through phosphorylation (…)”.
10 L392 typo rplI?	*R/We thank the reviewer for spotting this typo mistake.*<br><br>*The name of the gene has been corrected to “rplL” in fig. 4 and in its legend (line 477).*
