# Supplementary material for: The regulon of Brucella abortus two-component system BvrR/BvrS reveals the coordination of metabolic pathways required for intracellular life
Source: PLoS One. 2022 Sep 21;17(9):e0274397. doi: 10.1371/journal.pone.0274397 (PMC9491525; doi:10.1371/journal.pone.0274397)
Supplement: Attachment — Submitted filename: Response to reviewer.pdf [file pone.0274397.s008.pdf]

PROGRAMA DE INVESTIGACIÓN
EN ENFERMEDADES TROPICALES
Escuela de Medicina Veterinaria, Universidad Nacional,
Apartado Postal 304-3000, Heredia COSTA RICA
Fax: 506/22381298, Tel:506/22380761,
E-mail: catguz@una.cr

San José, August the 24th, 2022

Dear Editor:

Enclosed you will find a modified version of the manuscript ID: PONE-D-22-09403: “The regulon of *Brucella abortus* two-component system BvrR/BvrS reveals the coordination of metabolic pathways required for intracellular life”, in a clean and tracked changes formats.

We would like to thank the reviewer and editor for the helpful comments to improve the manuscript. Below you will find a table answering each of the reviewer’s comments. There is a change in Fig 3, consisting of adding a Venn diagram for a better representation of the data mining result, as Fig 3C. The data supporting such representation is now an additional sheet in Table S4.

Thank you for your consideration. Sincerely:

Caterina Guzmán Verri, PhD

Corresponding author

Responses to reviewer´s comments

Comment	Response
**Reviewer #2:** The authors misunderstood my request to define TCS, I meant to explain what the abbreviation means (even though is is defined in the abstract). I would add this to line 73, where they first introduce BvrRS. The additional text they added, to describe what a TCS is, is very good!	*Thanks for the clarification. We defined the abbreviation as requested in line 75 of the tracked version*
While the authors argue that confirming the BvrRS targets experimentally can wait for another study, they should mine the available transcriptomic and proteomic studies comparing WT and TCS mutants to see whether any of their predicted targets have been found. This will strengthen the manuscript.	*As requested, we performed data mining using the transcriptomic and proteomic studies with WT and TCS mutants. Detailed information is now presented as an additional excel sheet in table S4. Fig 3C was included and contains a Venn diagram presenting these results. Additionally, the following paragraphs were introduced in the results and discussion sections:*<br><br>*Line 379: "*Fig 3C compares the results of this study and those reporting putative BvrR/BvrS targets, using transcriptomic and proteomic analysis of *B. abortus* 2308 and *bvrR* mutant strains. The three studies converged on identifying four common target genes, while our study compared only to proteomics or transcriptomics presented respectively 20 and 15 additional common target genes (S4 Table)."<br><br>*Line 639 "*The BvrR binding sites described in this work should be considered *bonafide* putative gene regulation sites. Some of these regions have been previously identified as putative BvrR/BvrS targets [15,16] and deserve further investigation".
While the manuscript is understandable, the authors should have the text proof read by a native english speaker to correct the numerous small errors in grammar and syntax.	*We thank the reviewer for pointing this out. Indeed, there were numerous small errors in grammar and syntax. We have corrected as many as detected, as shown in the tracked version of the manuscript.*
